# RELATIONAL RELIGIOSITY, FORGIVENESS, AND FAMILY FUNCTIONING IN OLDER AFRICAN AMERICAN COUPLES

**DOI:** 10.1093/geroni/igae098.2554

**Published:** 2024-12-31

**Authors:** Antonius Skipper, Andrew Rose, Seungjong Cho

**Affiliations:** Georgia State University, Atlanta, Georgia, United States; Social Work, Lubbock, Texas, United States; Texas Tech University, Lubbock, Texas, United States

## Abstract

Despite recent advances in studies on positive psychology, relatively little is known about the factors that influence the romantic relationships of older African Americans. Existing literature does note that older African Americans are among the most religious racial/ethnic groups in the United States. In addition, forgiveness is recognized as a common mediator between relational religiosity and other positive outcomes, such as psychological well-being and successful aging. Given the need for more strength-focused studies on minority-based romantic relationships, this study examined forgiveness as a potential mediator between relational religiosity and family functioning among older, romantically involved African American couples. This study utilized the dyadic data of 194 African American couples with partners between the ages of 50 and 86 years. Data were captured with the Revised Sanctification of Marriage scale, six items assessing interpersonal forgiveness, and the McMaster Family Assessment Device. Indirect effects were verified with 95% confidence intervals within 10,000 bias-corrected bootstrap iterations. The relations between men’s relational religiosity and men’s reports of family functioning were partially mediated by men’s self-reported ability to forgive their partner (B =.053 (95% CI [.001,.187])), as well as the sum of all men’s and women’s reported abilities to forgive and perceive forgiveness (B =.132 (95% CI [.012,.260])). These findings highlight the growing, yet underrecognized, need to understand the religiosity of older African American men relative to positive relational outcomes. Further, this data helps to counter the deficit-narratives that often paint African American family functioning as poor and flawed.

